# Trends in bidi and cigarette smoking in India from 1998 to 2015, by age, gender and education

**DOI:** 10.1136/bmjgh-2015-000005

**Published:** 2016-04-06

**Authors:** Sujata Mishra, Renu Ann Joseph, Prakash C Gupta, Brendon Pezzack, Faujdar Ram, Dhirendra N Sinha, Rajesh Dikshit, Jayadeep Patra, Prabhat Jha

**Affiliations:** 1 Centre for Global Health Research, St. Michael's Hospital and Dalla Lana School of Public Health, University of Toronto, Toronto, Ontario, Canada; 2 Healis-Sekhsaria Institute of Public Health, Mumbai, Maharashtra, India; 3 International Institute of Population Studies, Mumbai, Maharashtra; 4 World Health Organization Regional Office of South East Asia, New Delhi, India; 5 Tata Memorial Hospital, Mumbai, Maharashtra, India

## Abstract

**Objectives:**

Smoking of cigarettes or bidis (small, locally manufactured smoked tobacco) in India has likely changed over the last decade. We sought to document trends in smoking prevalence among Indians aged 15–69 years between 1998 and 2015.

**Design:**

Comparison of 3 nationally representative surveys representing 99% of India's population; the Special Fertility and Mortality Survey (1998), the Sample Registration System Baseline Survey (2004) and the Global Adult Tobacco Survey (2010).

**Setting:**

India.

**Participants:**

About 14 million residents from 2.5 million homes, representative of India.

**Main outcome measures:**

Age-standardised smoking prevalence and projected absolute numbers of smokers in 2015. Trends were stratified by type of tobacco smoked, age, gender and education level.

**Findings:**

The age-standardised prevalence of any smoking in men at ages 15–69 years fell from about 27% in 1998 to 24% in 2010, but rose at ages 15–29 years. During this period, cigarette smoking in men became about twofold more prevalent at ages 15–69 years and fourfold more prevalent at ages 15–29 years. By contrast, bidi smoking among men at ages 15–69 years fell modestly. The age-standardised prevalence of any smoking in women at these ages was 2.7% in 2010. The smoking prevalence in women born after 1960 was about half of the prevalence in women born before 1950. By contrast, the intergenerational changes in smoking prevalence in men were much smaller. The absolute numbers of men smoking any type of tobacco at ages 15–69 years rose by about 29 million or 36% in relative terms from 79 million in 1998 to 108 million in 2015. This represents an average increase of about 1.7 million male smokers every year. By 2015, there were roughly equal numbers of men smoking cigarettes or bidis. About 11 million women aged 15–69 smoked in 2015. Among illiterate men, the prevalence of smoking rose (most sharply for cigarettes) but fell modestly among men with grade 10 or more education. The ex-smoking prevalence in men at ages 45–59 years rose modestly but was low: only 5% nationally with about 4 current smokers for every former smoker.

**Conclusions:**

Despite modest decreases in smoking prevalence, the absolute numbers of male smokers aged 15–69 years has increased substantially over the last 15 years. Cigarettes are displacing bidi smoking, most notably among young adult men and illiterate men. Tobacco control policies need to adapt to these changes, most notably with higher taxation on tobacco products, so as to raise the currently low levels of adult smoking cessation.

**Key questions**

**What is already known about this topic?**

- India has over 100 million adult smokers, the second highest number of smokers in the world after China.
- There are already about 1 million adult deaths per year from smoking.

**What are the new findings?**

- The age-standardised prevalence of smoking declined modestly among men aged 15–69 years, but the absolute number of male smokers at these ages grew from 79 million in 1998 to 108 million in 2015. This is due to population growth offsetting modest declines in prevalence.
- Cigarettes are displacing bidis, especially among younger men and among illiterate men. This change might further increase the smoker: non smoker relative risks of disease.
- Smoking cessation remains uncommon—only about 5% of men aged 45–59 years are ex-smokers. India has about 4 current male smokers for every quitter at these ages.
- Female smoking at ages 15–69 years has not likely risen.

**Recommendations for policy**

- More effective tobacco control policy including higher tobacco taxation for cigarettes needs to be implemented as a short-term priority. India's complicated tax structure has kept overall taxes on cigarettes low relative to other countries, with particularly low taxes on the less-expensive, short cigarettes that compete with the bidi market. Longer term policies need to raise taxes on bidis.
- Intervention programmes to raise the currently low levels of tobacco cessation are needed.
- Use of reliable, representative, large scale population surveys can help monitor the evolution of smoking and its consequences.

## Introduction

Tobacco smoking is among the largest preventable causes of premature deaths globally.^1^ In 2010, an estimated 120 million Indian adults smoked, making India second only to China in number of smokers.^2^ ^3^ Historically, most of the smoked tobacco in India has been in the form of bidis, small locally made cigarettes with tobacco wrapped inside a Tendu leaf. In 2010, smoking caused about 1 million deaths, or 10% of all deaths in India, with about 70% of these deaths occurring at the ages of 30–69 years.^4^ ^5^ The patterns of use of bidis or manufactured cigarettes vary across different regions and socioeconomic levels.^6^ ^7^ Smoking cessation is far less common than in high-income countries.^3^ ^4^ ^8^ ^9^

The consumption pattern of tobacco has likely changed over the last decade in response to substantially higher income in India paired with population growth and perhaps in response to modest tobacco control efforts.^10^ We examine three nationally representative surveys covering over 2.5 million homes and 14 million people to provide estimates of the changing trends from 1998 to 2015 in any tobacco smoking, in bidi and cigarette smoking, and in smoking cessation. We examine these trends by gender, age and generation, education level, and urban and rural residence.

## Methods

### Survey populations

We estimated smoking prevalence using three large, nationally representative surveys in India: the Special Fertility and Mortality Survey (SFMS) (1998), Sample Registration System Baseline Survey (SRSBS) (2004) and the Global Adult Tobacco Survey (GATS) (2010).

The Registrar General of India (RGI) divides India into small geographic areas called Sample Registration System (SRS) units, based on the preceding census, to estimate vital rates nationally and for each state. In 1993, the RGI randomly selected 6671 of these small areas (4436 rural and 2235 urban) from the 1991 census to form the 1993–2003 SRS frame. The SFMS was undertaken in these SRS units starting in February 1998, covering approximately 6 million people living in approximately 1.1 million households across India. Jammu and Kashmir and the rural units of Nagaland (<1% of India's population) were excluded due to operational problems.^11^ In 2004, the RGI randomly selected 7597 areas (4433 rural and 3164 urban) from the 2001 census, to form the 2004–2013 SRS frame. The SRSBS was conducted over several months in 2004, covering 1.3 million households in all Indian states and union territories.^12^ In 2010, the GATS surveyed over 79 690 nationally representative households selected from the 2001 census enumeration blocks and villages for urban and rural areas, respectively. The GATS covered all non-institutionalised adults within 29 states and two union territories (Chandigarh and Puducherry). A total of 69 296 adults completed the interview and provided self-reported information on their smoking habits.^3^ The survey design and sample framework details, field methods and quality control steps for each survey have been published.^3^ ^11–13^ We applied Indian census definitions for education (illiterate or with no formal education, less than grade 10 education and grade 10 or more education), and for rural and urban residency status.

### Definitions of smoking

Most of the respondents in the 1998 SFMS and the 2004 SRSBS were male heads of the household and provided personal information as well as proxy information on other household members, including their wives. The SFMS asked respondents if household members aged 10 years or older were current smokers (combining occasional and daily smokers); and if yes, whether they smoked cigarette or bidis. The SRSBS asked respondents if persons aged 15 years or older were usual smokers, occasional smokers, ex-smokers or never-smokers; we defined current smokers as usual or occasional smokers. The 2010 GATS randomly selected one individual respondent from each household and asked them to categorise themselves into daily smokers, less than daily smokers, ex-smokers or never-smokers; we defined current smokers as daily or as less than daily smokers. The SRSBS did not publish information on type of tobacco smoked, and reported statistics only for age groups 15–29, 30–44, 45–59 and 60 years or older. Ex-smoking was reported only in the 2010 GATS and in the 2004 SRSBS, the latter only for any smoking. Respondents who smoked cigarettes or bidis exclusively were classified as ‘exclusive’ while respondents who smoked both cigarettes and bidis were classified as ‘both’. We also included ‘any smokers’ as a separate category for those who were either exclusive or both or smoked any other type of tobacco including hookah, cheroot, etc.^14^

### Analysis

We analysed all states and union territories but retained three states formed after the 1991 census within their original states (thus, Madhya Pradesh and Chhattisgarh, Bihar and Jharkhand, and Uttar Pradesh and Uttarakhand, are listed as pairs). We combined the smaller states of the Northeast (except Assam) to include Arunachal Pradesh, Manipur, Meghalaya, Mizoram, Nagaland, Sikkim and Tripura. Sample framework and weights were used to derive prevalence estimates. Analyses were conducted in STATA V14.0.

We standardised smoking prevalence to the age distribution of the 2011 Indian Census.^15^ We tested statistical significance of the differences between the surveys by age-standardised rate ratios (ASRRs) with 99% CIs. Estimates of the absolute number of smokers for both genders in 1998 applied the survey age-specific prevalence to the interpolated population totals from the 1991 to 2001 censuses. Estimates of the absolute number of smokers in 2015 applied annual rates of change in smoking prevalence between 1998 and 2010, for each age and state strata for men and 15–69 years for women, to the 2015 United Nations population totals.^16^ Throughout, we focus on smoking at ages 15–69 years for three reasons. First, smoking trends among ages 70 years or older are less reliably ascertained (due in part to higher absolute mortality rates from smoking at older ages).^17^ Second, India's age structure is still quite young, with 80% of India's population being below 45 years of age.^16^ Third, about 70% of tobacco deaths in India are reported at ages 30–69 years.^4^ Hence, the more important public health goal is avoidance of death in middle versus older age.^18^

## Results

We were able to report on men and women in 29 Indian states representing 99% of India's population (Jammu and Kashmir, and 7 of the 8 union territories were excluded from 1 or 2 of the surveys). Individual level data on smokers analysed about 530 000 men and 32 000 women above age 15 years in 1998, and 10 000 men and 1200 women in 2010 (see web table 1).

10.1136/bmjgh-2015-000005.supp1 Supplementary tables

**Figure 1:**
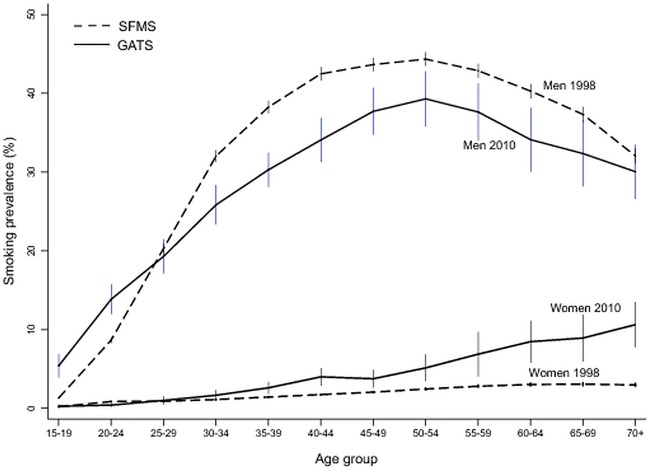
Smoking prevalence by age and gender (with 99% CI): 1998–2010. GATS, Global Adult Tobacco Survey; SFMS, Special Fertility and Mortality Survey.

The age-specific prevalence of any smoking in men followed a similar pattern in 1998 and 2010, peaking at about 50 years of age and declining at older ages. At most ages, the age-specific prevalence was significantly lower in 2010 than in 1998 (figure 1). The notable exception was in men aged 15–29 years, among whom the age-specific prevalence was higher in 2010 than in 1998. The age-specific prevalence of any smoking in women showed a very different pattern than that for men, with prevalence peaking above 60 years of age. The observed increases in the age-specific prevalence in women between the 1998 and 2010 surveys were concentrated mostly at these older ages. Age-specific prevalence rates in men in 2004 for any smoking at ages 15–29, 30–44 and 45–59 years, were in between the 1998 and 2010 results (see web table 2A–C), consistent with modest increases at ages 15–29 years and modest declines in the latter two age groups.

**Figure 2:**
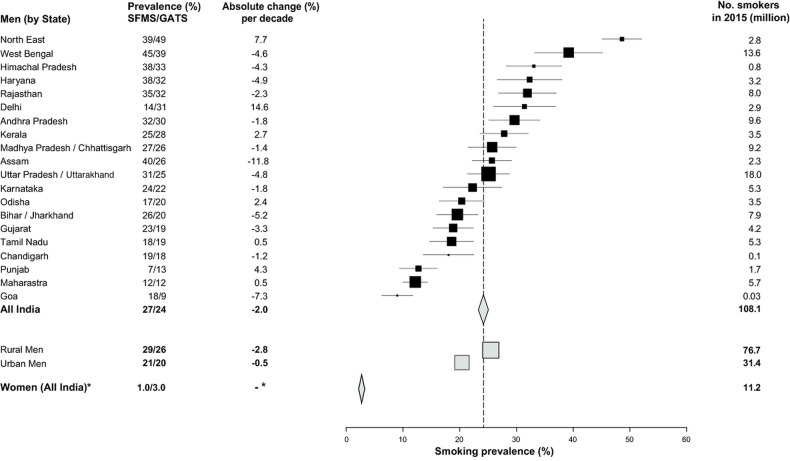
Age-standardised smoking prevalence among adults aged 15-69 years, by state: 1998–2010, absolute change (%) per decade and number of smokers in 2015 (in millions). GATS, Global Adult Tobacco Survey; SFMS, Special Fertility and Mortality Survey. Note that reliable estimates for absolute changes per decade could not be estimated for women. We applied GATS 2010 smoking prevalence for women ages 15-69 years to the 2015 population.

**Figure 3:**
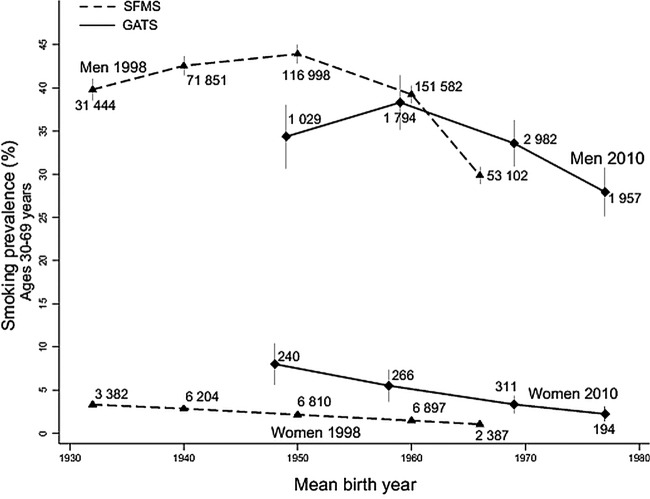
Smoking prevalence by decade of birth cohort and gender: 1998–2010. Note: Numbers near each point represent the sample size. GATS, Global Adult Tobacco Survey; SFMS, Special Fertility and Mortality Survey.

The age-standardised prevalence of any smoking in men aged 15–69 years fell from about 27% in 1998 to 24% in 2010 (change per decade −2.0%, ASRR=0.9, 99% CI 0.9 to 0.9; figure 2). The age-standardised prevalence of any smoking in women aged 15–69 years rose from 1.4% in 1998 to 2.7% in 2010, but this might well be an artefact of the survey methodology as most of the reported increase occurred at older ages. The SFMS of 1998^12^ ^13^ ^19^ relied on proxy reporting by male heads of household and they likely under-reported female smoking,^20–22^ whereas the smoking prevalence was more accurately captured from self-reports in the GATS of 2010.^3^ The changes between 1998, 2004 and 2010 did not show consistent increases in smoking in young adult women, as would be expected if there were an increase in female smoking. In each time period, smoking prevalence at ages 30–69 years in women born after 1960 was about half of the prevalence in women born before 1950. By contrast, the intergenerational changes in smoking prevalence in men at these ages were much smaller (figure 3). As few women smoked, most of the following results focus on men.

**Figure 4:**
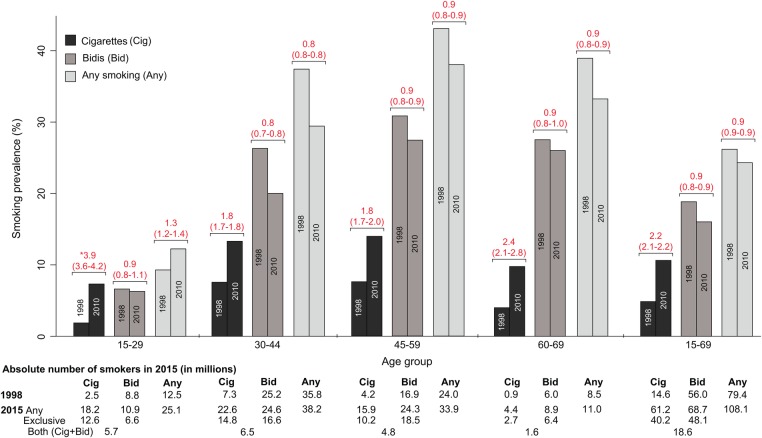
Age-standardised rate (ASR) of smoking among men by age group, product between 1998 to 2010 and number of smokers in 2015 (in millions). ASRR, age-standardised rate ratios between the Global Adult Tobacco Survey and the Special Fertility and Mortality Survey.

The age-standardised prevalence of any smoking among men aged 15–69 years rose significantly in Delhi, Kerala, Odisha, Punjab and the Northeast States but fell modestly in other states (figure 2). The age-standardised prevalence of any smoking in men aged 15–29 years rose (9–12%; change per decade +2.4%, ASRR=1.3, 1.2 to 1.4), but fell at older ages. These trends markedly diverged between cigarettes and bidis (figure 4). Cigarette smoking became more prevalent at ages 15–69 years (change per decade +4.8%, ASRR 2.2, 2.1 to 2.2) with large increases at ages 15–29 years (change per decade +4.5%, ASRR 3.9, 3.6 to 4.2) and smaller, but still significant, increases at older ages. By contrast, the age-standardised prevalence of bidi smoking among men fell at ages 15–69 years (change per decade −2.3%, ASRR 0.9, 0.8 to 0.9), and within each age group. Even using the GATS definition of exclusive smoking, cigarette smoking rose substantially and bidi smoking fell modestly (data not shown). Cigarette smoking rose more in rural men than in urban men while bidi smoking fell modestly in both areas for nearly all age groups except at ages 15–29 years (see web figures 1A,B).

10.1136/bmjgh-2015-000005.supp2 Supplementary figures

**Figure 5:**
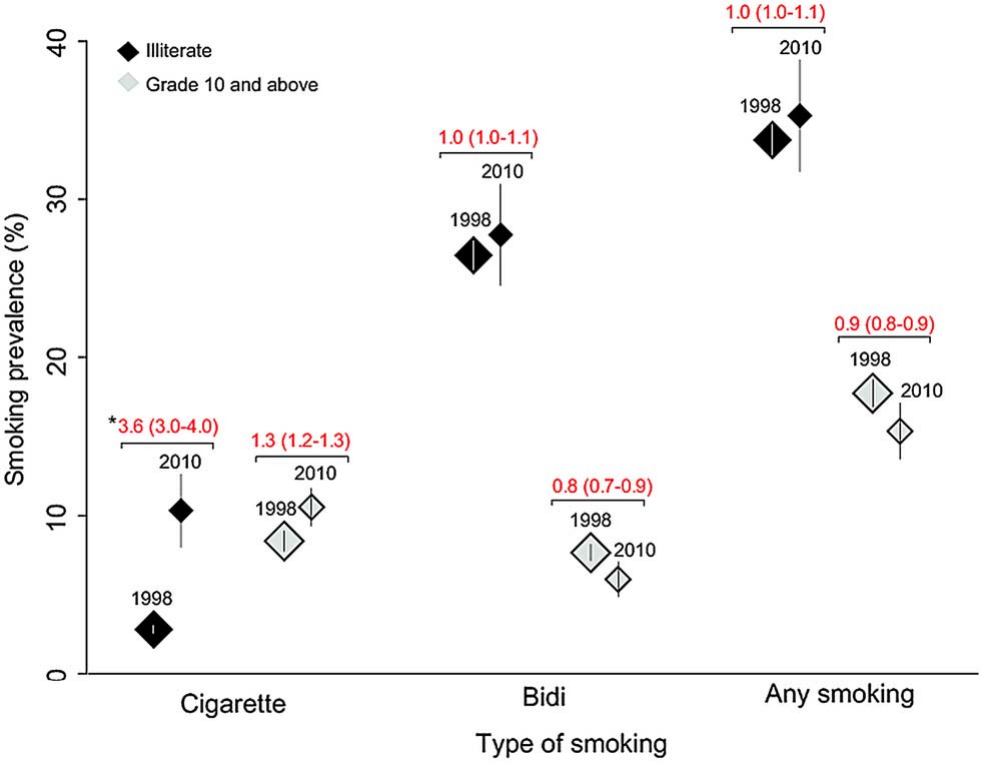
Age-standardised rate (ASR) of smoking among men aged 15 to 69 years by product, and by two levels of education: 1998–2010. ASRR, age-standardised rate ratios between the Global Adult Tobacco Survey and the Special Fertility and Mortality Survey.

The highest age-standardised prevalence of any smoking in men aged 15–69 years was in illiterate men in both 1998 and 2010 (see web table 3). Among illiterate men at these ages, the prevalence of cigarette, bidi or any smoking rose (figure 5), most sharply for cigarettes. By contrast, among men with grade 10 or more education, the prevalence of bidi or any smoking fell. Even among these more educated men, cigarette smoking rose modestly.

As the age-specific rates in 1998, 2004 and 2010 showed consistent increases at ages 15–29 years and modest declines at older ages, we were able to make reasonably sensible forward projections to 2015. The absolute numbers of men smoking any type of tobacco at ages 15–69 years rose by about 29 million or 36% in relative terms from 79 million in 1998 to 108 million in 2015 (figure 4, bottom panel), representing an average annual increase of about 1.7 million male smokers. The number of any smokers at these ages rose in urban India by about 68% from 19 to 31 million and the number in rural India increased about 26% from 61 to 77 million. The five largest relative increases over one decade in the absolute number of male smokers aged 15–69 years were in Delhi (220%), Punjab (120%), the North East states excluding Assam (60%), Odisha (50%) and Maharashtra (40%). Decreases in the absolute number of male smokers were observed in only two states—Goa and Assam (see web table 1). In 2015, we estimate that approximately 61 million Indian adult men aged 15–69 years smoked cigarettes (40 million exclusively) and 69 million smoked bidis (48 million exclusively). At ages 15–69 years, and assuming that the prevalence in GATS in 2010 was unchanged by 2015, there were about 11 million women who smoked, about one-tenth the total of men who smoked. Given higher mortality and greater reporting problems at older ages, there were, less reliably, an estimated 6 million men and 2.5 million women smokers at ages 70 years and above.

**Figure 6:**
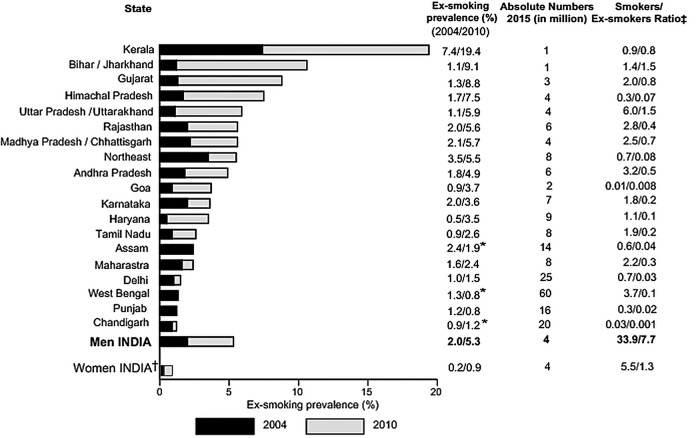
Ex-smoker rates in India among men (45–59 years) from 2004 to 2010; absolute number (in million) of smokers and ex-smokers in 2015. GATS, Global Adult Tobacco Survey; UN, United Nations. Note that, * it is unlikely that smoking cessation has decreased from 2010-2015. Therefore we assigned GATS 2010 ex-smoking prevalence to Assam, West Bengal and Punjab which reported a decline. Odisha was excluded from the analyses due to few numbers of smokes in the GATS. †We applied GATS 2010 smoking and ex-smoking prevalence for women to estimate 2015 absolute totals. ‡The number of smokers and ex-smokers are based on 2015 UN Population totals for India.

Smoking cessation among men is best measured at ages 45–59 years, well after the peak of smoking initiation and when smokers are more likely to be aware of health hazards that could prompt cessation.^17^ The age-specific ex-smoking prevalence in men at these ages rose modestly from 2004 to 2010, from about 2% to 5% nationally (figure 6), with variation in the ex-smoking prevalence across states in 2010 ranging from 19% in Kerala to <1% in Punjab. The ratio of current to former smokers in men at these ages was 4 in 2015 for India, ranging from 1 in Kerala and Bihar/Jharkhand to 60 in West Bengal. Very few women were ex-smokers.

## Discussion

The Indian government implemented the Cigarettes and Other Tobacco Products Act (COTPA)^23^ in 2003 and ratified the WHO's Framework Convention on Tobacco Control in 2004, as well as the Cable Television Networks (Amendment) Act 2000 prohibiting tobacco advertising in all state-controlled electronic media and publications, including cable television.^24^ ^25^ Further, the Government has also included tobacco control in the priorities of the ongoing National Rural Health Mission.^26^ Despite these programmes, the major challenge to success is effective implementation of the provisions of COTPA, especially in enforcement of bans on smoking in public places (which are known to raise cessation rates).^1^ ^24^ Most importantly, these trends in smoking reflect the lack of substantial increases in tobacco excise taxes, which have not kept up with the increased affordability of cigarettes and bidis.^10^ Hence, tobacco control in India urgently requires effective implementation of national policies.

Our study of nationally representative Indian surveys over more than a decade finds substantial increases in the number of male smokers aged 15–69 years, rising over one-third since 1998 to nearly 108 million in 2015. The increase is mostly due to population growth offsetting the modest declines in prevalence over this time period, similar to the pattern observed in other countries.^27^ There is a clear shift in consumption away from bidis towards manufactured cigarettes. The sharpest relative and absolute increase was for cigarette smoking, particularly in young adult men aged 15–29 years. The increases in cigarette use among younger adult men were seen in rural areas and were greatest among illiterate men.

Rapid income growth over the last decade has most likely contributed to the shift in smoking—from the less-expensive bidis to cigarettes. Price is the most important determinant of consumption. Relative to income, cigarettes and bidis have become less costly in the last decade.^10^ Moreover, and most relevant for policy, India's complicated tax structure has kept overall taxes on cigarettes low relative to other countries, with particularly low taxes on the inexpensive, short cigarettes that compete with the bidi market.^28–30^ The increase in cigarette smoking is consistent also with market reports showing that the absolute volume of cigarettes sold in India has risen from about 98 billion sticks in 2000 to 114 billion in 2012.^31^ Unfortunately, bidi sales data are unavailable, as most bidis are sold by small cottage industries with little monitoring or regulation and attract low or no taxes.^32^

The observed increase in female smoking is likely to be an artefact of reporting. A true increase in smoking would be expected among younger women, who have seen more rapid income growth, and are the subject of tobacco industry promotion. However, among younger adult women, there was little increase in smoking (figure 1, see web table 1), and indeed smoking prevalence was less than half of that seen in older generations. Among female respondents in the ongoing Million Death Study (conducted in the same areas as the SRSBS), there has been no major shift in smoking patterns among younger women from 2004 to 2013 (data not shown). Among selected high schools in particular regions of India, there are reports that female and male smoking at very young ages (13–15 years) are more equal,^33^ and, in the future, young women, particularly in urban areas, could take up cigarette smoking.

A robust measure of successful tobacco control policies is rapid increases in ex-smoking prevalence among later age adults.^17^ ^34^ We find that smoking cessation remains uncommon in India: only about 5% of the men aged 45–59 years were ex-smokers in 2010, a modest increase from 2004. A similarly helpful statistic is the ratio of current to former smokers. India has about four current male smokers for every quitter at these ages. By contrast, in the USA and other high-income countries, there are now as many ex-smokers as current smokers at these ages.^35^ ^36^ Countries that are more comparable to India in income but adopted tobacco control earlier (including through tax increases) now have a substantially higher prevalence of quitting.^37^

The absolute number of tobacco-attributable deaths among India's 120 or so million smokers will continue to rise. The exact totals depend on the risks of smoking cigarettes or bidis, the age of initiation and background mortality rates from causes other than smoking. Indian male smokers traditionally start smoking later in life^4^ and smoke fewer sticks per day when compared with male smokers in high-income countries.^9^ Nonetheless, the smoker versus non-smoker risk ratios for dying from smoking attributable causes remain high in India.^4^ Unlike in China,^38^ we did not see a dramatic shift to smoking at younger ages (data not shown) among men. Some, but not all, studies have shown that the relative risk of tobacco attributable causes of death is greater for cigarette than for bidi smoking.^4^ ^14^ The risks of death among cigarette smokers who smoke more than eight cigarettes per day, closer to smoking patterns in the USA or Canada, are particularly pronounced.^1^ ^39^ Future risks will also depend on whether the currently low quit rates rise substantially.

Our analyses need to consider a few limitations. First, the SFMS and the SRSBS relied on proxy reporting by the head of the household, which has shown in some direct comparisons to be valid^20–22^ and tightly correlated with self-reports. However, other studies find that the correlation between proxy and self-responses varies,^40^ including by gender or relationship status of the respondent and subject.^41^ The reported increase in female smoking prevalence at older ages between the 1998 SFMS or 2004 SRSBS and the 2010 GATS suggests a reporting bias from husbands unaware of or unwilling to disclose their wife's smoking. Proxy reporting might also result in under-reporting of smoking at younger ages. However, other surveys^42^ ^43^ find prevalence fits with increased consumption among younger adult men.

Second, whereas the GATS allowed smokers to self-identify smoking multiple products, the SFMS required classifying smokers as either cigarette smokers or bidi smokers. Thus, we might have underestimated cigarette and bidi smoking prevalence rates in the 1998 baseline, and exaggerated the actual increase by 2010. Such misclassification of cigarette or bidi smoking would not affect the findings for any smoking prevalence. Moreover, smoking prevalence among living respondents in the ongoing Million Death Study finds also an increased use of cigarettes relative to bidis. Lastly, the sampling frame for the surveys differed, being much larger for SFMS and SRSBS than GATS, and the GATS method of selecting households may have missed (the few) adults living alone.^3^ All three sampling frames were nationally representative, with large sizes, and likely accurately captured the true underlying trends in male smoking.

A substantial reduction in smoking is central to achieving the United Nations’ 2030 goals to decrease premature death from non-communicable disease.^18^ Apart from effective implementation of the WHO's recommended measures, including increasing tobacco taxation,^1^ the use of reliable, representative, large scale population surveys is helpful in accelerating tobacco control policies and in monitoring their impact, particularly in raising the low rates of smoking cessation in India.

## References

[1] Jha P, Peto R Global effects of smoking, of quitting, and of taxing tobacco. N Engl J Med 2014;370:60–8. 10.1056/NEJMra1308383 24382066

[2] Shafey O, Eriksen M, Ross H The Tobacco Atlas. 3rd edn Atlanta, USA: American Cancer Society, 2009.

[3] Global Adult Tobacco Survey: India Report. Mumbai, India: International Institute for Population Sciences (IIPS), Ministry of Health and Family Welfare GoI, 2010 http://mohfw.nic.in/WriteReadData/l892s/1455618937GATS%20India.pdf

[4] Jha P, Jacob B, Gajalakshmi V A nationally representative case-control study of smoking and death in India. N Engl J Med 2008;358:1137–47. 10.1056/NEJMsa0707719 18272886

[5] Gajalakshmi V, Peto R, Kanaka TS Smoking and mortality from tuberculosis and other diseases in India: retrospective study of 43000 adult male deaths and 35000 controls. Lancet 2003;362:507–15. 10.1016/S0140-6736(03)14109-8 12932381

[6] Rani M, Bonu S, Jha P Tobacco use in India: prevalence and predictors of smoking and chewing in a national cross sectional household survey. Tob Control 2003;12:e4 10.1136/tc.12.4.e4 14660785 PMC1747786

[7] Subramanian SV, Nandy S, Kelly M Patterns and distribution of tobacco consumption in India: cross sectional multilevel evidence from the 1998–9 national family health survey. BMJ 2004;328:801–6. 10.1136/bmj.328.7443.801 15070637 PMC383376

[8] Gupta PC Survey of sociodemographic characteristics of tobacco use among 99,598 individuals in Bombay, India using handheld computers. Tob Control 1996;5:114–20. 10.1136/tc.5.2.114 8910992 PMC1759496

[9] Jha P, Ranson MK, Nguyen SN Estimates of global and regional smoking prevalence in 1995, by age and sex. Am J Public Health 2002;92:1002–6. 10.2105/AJPH.92.6.1002 12036796 PMC1447501

[10] Jha P, Guindon GE, Joseph RA A rational taxation system of bidis and cigarettes to reduce smoking deaths in India. Econ Polit Wkly 2011;42:44–51.

[11] Special Fertility and Mortality Survey 1998: a report on 1 million homes. New Delhi, India: Registrar General of India, 2005.

[12] Sample registration system, baseline report of 2004. New Delhi, India: Registrar-General of India, 2007.

[13] Sample Registration System: Statistical Report 2013. New Delhi, India: Registrar General of India, 2014.

[14] Gupta PC, Pednekar MS, Parkin DM Tobacco associated mortality in Mumbai (Bombay) India. Results of the Bombay Cohort Study. Int J Epidemiol 2005;34:1395–402. 10.1093/ije/dyi196 16249218

[15] India Census Data 2011. The Registrar General & Census Commissioner, India, New Delhi, Ministry of Home Affairs: Government of India http://www.censusindia.gov.in/

[16] World Population Prospects Database extract: United Nations, Department of Economic and Social Affairs, Population Division, 2015 http://esa.un.org/unpd/wpp/Download/Standard/Population/

[17] Jha P Avoidable global cancer deaths and total deaths from smoking. Nat Rev Cancer 2009;9:655–64. 10.1038/nrc2703 19693096

[18] Norheim OF, Jha P, Admasu K Avoiding 40% of the premature deaths in each country, 2010–30: review of national mortality trends to help quantify the UN sustainable development goal for health. Lancet 2015;385:239–52. 10.1016/S0140-6736(14)61591-9 25242039

[19] Fu SH, Jha P, Gupta PC Geospatial analysis on the distributions of tobacco smoking and alcohol drinking in India. PLoS ONE 2014;9:e102416 10.1371/journal.pone.0102416 25025379 PMC4099149

[20] Gilpin EA, Pierce JP, Cavin SW Estimates of population smoking prevalence: self-vs proxy reports of smoking status. Am J Public Health 1994;84:1576–9. 10.2105/AJPH.84.10.1576 7943473 PMC1615093

[21] Hyland A, Cummings KM, Lynn WR Effect of proxy-reported smoking status on population estimates of smoking prevalence. Am J Epidemiol 1997;145:746–51. 10.1093/aje/145.8.746 9126001

[22] Mohan D, Neufeld K, Chopra A Agreement between head of household informant and self-report in a community survey of substance use in India. Drug Alcohol Depend 2003;69:87–94. 10.1016/S0376-8716(02)00247-8 12536069

[23] The Cigarettes and Other Tobacco Products (Prohibition of Advertisement and regulation of Trade and Commerce, Production, Supply and Distribution) Act; An Act enacted by the Parliament of Republic of India by notification in the Official Gazette. (Act 32 of 2003). In: India Po, ed. New Delhi, 2003 http://www.who.int/fctc/reporting/Annexthreeindia.pdf

[24] WHO. Framework convention on tobacco control. Geneva: World Health Organization, 2004.

[25] The Cable Television Networks (Amendment) Act In: India Go, ed., 2000 http://www.wipo.int/edocs/lexdocs/laws/en/in/in033en.pdf

[26] Kaur J, Jain DC Tobacco control policies in India: implementation and challenges. Indian J Public Health 2011;55:220–7. 10.4103/0019-557X.89941 22089690

[27] Ng M, Freeman MK, Fleming TD Smoking prevalence and cigarette consumption in 187 countries, 1980–2012. JAMA 2014;311:183–92. 10.1001/jama.2013.284692 24399557

[28] Bahri C How India's tax system helps heavily taxed cigarettes flourish, Mumbai, India: India Spend, September, 2015 10.1136/bmjgh-2015-000005

[29] Bahri C Why India ignores a $16-billion smoking-led health crisis. Mumbai, India: India Spend, September, 2015 http://www.indiaspend.com/cover-story/why-india-ignores-a-16-billion-smoking-led-health-crisis-37620

[30] Guindon GE, Mishra S, Jha P Still ample room to raise India's tobacco tax. BMJ 2014;349:g4.680.

[31] Cigarettes in India. A market report from ERC. The Canadean Group, 2014.

[32] Nandi A, Ashok A, Guindon GE Estimates of the economic contributions of the bidi manufacturing industry in India. Tob Control 2015;24:369–75. 10.1136/tobaccocontrol-2013-051404 24789606

[33] Sinha DN, Gupta PC, Gangadharan P Tobacco use among students and school personnel in India. Asian Pac J Cancer Prev 2007;8:417–21. 18159980

[34] Jha P, Chaloupka FJ, Moore J Tobacco addiction. In: Jamison DT, Breman JG, Measham AR, eds Disease control priorities in developing countries. 2nd edn Washington DC: World Bank; 2006 445–493. 21250309

[35] Jha P, Ramasundarahettige C, Landsman V 21st-century hazards of smoking and benefits of cessation in the United States. N Engl J Med 2013;368:341–50. 10.1056/NEJMsa1211128 23343063

[36] Jha P, MacLennan M, Yurekli A Global hazards of tobacco and the benefits of smoking cessation and tobacco taxes in Cancer. Gellband H, Jha P, eds. Disease Control Priorities. 3rd Edition 2015:175–93. 26913345

[37] Giovino GA, Mirza SA, Samet JM Tobacco use in 3 billion individuals from 16 countries: an analysis of nationally representative cross-sectional household surveys. Lancet 2012;380:668–79. 10.1016/S0140-6736(12)61085-X 22901888

[38] Chen Z, Peto R, Zhou M Contrasting male and female trends in tobacco-attributed mortality in China: evidence from successive nationwide prospective cohort studies. Lancet 2015;386:1447–56. 10.1016/S0140-6736(15)00340-2 26466050 PMC4691901

[39] Jha P, Gupta PC, Peto R Case–control study of smoking and death in India. N Engl J Med 2008;358:2842–5. 18579823 10.1056/NEJMc080813

[40] Navarro AM Smoking status by proxy and self report: rate of agreement in different ethnic groups. Tob Control 1999;8:182–5. 10.1136/tc.8.2.182 10478403 PMC1759697

[41] Harakeh Z, Engels RC, Vries HD Correspondence between proxy and self-reports on smoking in a full family study. Drug Alcohol Depend 2006;84:40–7. 10.1016/j.drugalcdep.2005.11.026 16386380

[42] National Family Health Survey (NFHS3) 2005–06. In: Ministry of Health and Family Welfare GoI, and International Institute for Population Sciences. India, ed. Mumbai, 2007 https://dhsprogram.com/pubs/pdf/FRIND3/FRIND3-Vol1AndVol2.pdf

[43] Causes of death in India, 2001–03: Sample Registration System. Registrar General of India & Centre for Global Health Research 2009 http://www.censusindia.gov.in

