# Supplementary material for: Trends in bidi and cigarette smoking in India from 1998 to 2015, by age, gender and education
Source: BMJ Glob Health. 2016 Apr 6;1(1):e000005. doi: 10.1136/bmjgh-2015-000005 (PMC5321300; doi:10.1136/bmjgh-2015-000005)
Supplement: Supplementary tables [file bmjgh-2015-000005supp_tables.pdf]

Supplementary Appendix

Web Table 1: Number of smokers in India (sample and population): 1998 and 2010.

	Number of smokers studied	Number of smokers (000)		
**State and Region**	**1998/2010**	**1998**	**2015**	**Absolute change per decade(%)**
**Men 15-69 Years**				
Himachal Pradesh	10,998/360	674	839	14
Punjab	4095/160	560	1700	120
Chandigarh	2175/204	64	84	19
Haryana	24,254/364	2291	3231	24
Delhi	4815/328	612	2851	215
Rajasthan	34,952/370	5286	8035	31
Uttar Pradesh & Uttaranchal	54,445/821	14,523	18,014	14
Bihar & Jharkhand	38,646/381	7413	7855	4
Assam	28,840/713	3044	2340	-14
West Bengal	67,614/652	11,058	13,582	13
Odisha	15,667/230	1878	3488	50
Madhya Pradesh & Chhattisgarh	31,865/479	6168	9165	29
Gujarat	20,659/258	3443	4242	14
Maharashtra	12,906/326	3441	5707	39
Andhra Pradesh	30,599/416	7323	9605	18
Karnataka	27,452/217	3863	5333	22
Goa	3924/84	76	33	-33
Kerala	21,462/230	2270	3534	33
Tamil Nadu	20,636/268	3575	5315	29
Northeast	44,693/2379	1386	2796	60
Rural Men	41,2042/6449	60,957	76,718	15
Urban Men	96,965/3321	18,746	31407	40
INDIA TOTAL	**509,007/9770**	79,428	108,125	21
**Total 70+**	21,864/478	4128	5896	25
**Total 15+**	530,871/10248	83,437	114,021	22
**Women 15-69 Years INDIA**	**31,949/1214**		**11,183**	**-***

*Note: * Reliable estimates for annual absolute change (%) for women could not be estimated since we believe that smoking among women has been declining based on our estimation by birth cohorts.*

Web Table 2a: The 1998 and 2010 age standardized smoking prevalence for ages 15-69 years and 15-29 years, by state and location of residence and the 2004 non-standardized rates of smoking for 15-29 years[*].

States/ Regions	15-69 years						15-29 years								
	Total		Rural		Urban		Total			Rural			Urban		
	1998	2010	1998	2010	1998	2010	1998	2004	2010	1998	2004	2010	1998	2004	2010
**Men**															
Himachal Pradesh	38.2	33.1	38.2	33.1	38	33.1	12.1	8.0	13.7	12.2	7.8	12.7	11.0	8.6	19.6
Punjab	7.5	12.7	7.5	12.8	7.5	12.7	3.0	5.4	8.3	2.6	4.8	5.4	3.7	7.0	12.6
Chandigarh	19.4	18.0	18.9	16.5	19.4	18.1	11.4	11.2	9.6	21.9	17.8	19.3	9.9	7.5	8.3
Haryana	38.2	32.3	37.9	32.0	38.8	32.9	16.0	17.2	12.7	19.2	20.6	14.3	7.4	10.8	9.3
Delhi	13.9	31.4	13.5	31.0	13.9	31.4	4.8	10.3	20.8	6.5	10.5	9.8	4.7	10.3	21.1
Rajasthan	34.7	31.9	34.6	31.7	35.1	32.4	14.3	21.4	12.8	16.0	24.7	13.2	9.0	5.8	11.5
Uttar Pradesh/ Uttaranchal	30.8	25.1	30.8	25.1	30.6	24.9	10.0	13.8	9.9	10.6	15.2	11.0	7.9	8.3	6.0
Bihar/ Jharkhand	25.8	19.6	25.8	19.6	25.6	19.5	7.9	12.0	16.0	8.3	14.1	16.8	5.7	6.8	11.2
Assam	39.8	25.7	39.4	25.5	42.0	26.6	15.0	18.9	15.4	15.7	23.0	14.2	10.7	16.0	21.1
West Bengal	44.7	39.2	44.0	38.8	46.2	40.1	20.7	19.8	23.9	21.7	21.5	23.8	18.0	15.3	24.1
Odisha	17.5	20.4	17.5	20.3	17.4	20.6	6.0	11.7	10.1	6.5	12.7	10.1	3.8	8.2	11.0
Madhya Pradesh/ Chhattisgarh	27.4	25.7	27.3	25.7	27.5	25.8	8.8	12.8	16.2	10.2	16.7	15.4	5.1	5.3	19.6
Gujarat	22.8	18.8	22.9	19.0	22.6	18.7	7.5	9.5	3.7	6.9	10.9	3.5	8.3	5.4	4.0
Maharashtra	11.6	12.2	11.6	12.2	11.6	12.1	2.6	2.8	4.8	2.7	2.0	3.5	2.5	4.2	6.1
Andhra Pradesh	31.8	29.7	31.8	29.8	31.7	29.3	11.0	8.4	12.0	12.3	8.8	11.6	7.7	7.0	12.9
Karnataka	24.4	22.3	24.4	22.3	24.3	22.3	6.8	7.1	9.6	6.7	6.9	8.7	6.8	7.9	10.8
Goa	17.8	9.0	18.1	9.0	17.7	8.9	2.3	2.3	4.0	2.3	2.4	4.4	2.2	2.2	3.7
Kerala	24.6	27.8	24.5	27.8	24.7	27.9	6.9	9.0	16.6	7.7	9.7	14.6	4.7	5.8	22.6
Tamil Nadu	18.0	18.6	17.7	18.3	18.4	18.8	5.2	5.3	7.2	4.7	5.2	6.0	5.9	5.5	8.4
North East	39.4	48.6	39.0	48.4	40.2	49.1	17.6	21.7	34.9	18.2	22.0	34.5	16.0	20.3	35.9
INDIA TOTAL	26.7	24.3	29.3	26.0	21.0	20.4	9.4	11.8	12.3	10.4	13.0	12.8	7.0	8.8	11.1
**Women**															
INDIA TOTAL	1.4	2.7	1.6	3.3	0.9	0.4	0.6	1.3	0.5	0.7	1.5	0.7	0.5	0.8	0.2

[*] *Note: The 2004 estimates are age-specific prevalence estimates from SRSBL. SRSBL provided appropriate age specific patterns that are close to 2011 census population which is used as the standard population for age standardized rates using SFMS and GATS.*

Web Table 2b: The 1998 and 2010 age standardized smoking prevalence for ages 30-44 years and 45-59 years, by state and location of residence and the 2004 non-standardized rates of smoking at these ages*.

	30-44 years									45-59 years								
	Total			Rural			Urban			Total			Rural			Urban		
**States/ Regions**	**1998**	**2004**	**2010**	**1998**	**2004**	**2010**	**1998**	**2004**	**2010**	**1998**	**2004**	**2010**	**1998**	**2004**	**2010**	**1998**	**2004**	**2010**
**Men**																		
Himachal Pradesh	51.1	38.1	40.2	52.0	41.9	40.9	45.5	30.9	34.6	62.6	47.7	57.3	64.6	54.9	60.2	49.4	34.3	37.5
Punjab	10.5	15.1	17.0	9.7	13.3	16.9	11.7	19.0	17.3	11.6	15.7	12.3	11.3	13.7	11.6	12.1	20.5	12.7
Chandigarh	28.6	28.0	20.5	45.6	31.0	27.1	26.5	26.8	19.6	23.5	26.0	28.2	35.0	28.0	47.2	22.7	25.5	26.4
Haryana	53.7	47.5	42.2	62.9	56.9	44.2	35.1	32.3	38.7	60.0	51.4	55.5	70.7	63.0	58.3	40.6	34.1	49.4
Delhi	20.3	29.6	35.4	26.6	33.6	63.1	20.0	29.1	34.7	23.0	34.5	37.9	31.4	46.4	71.6	22.6	33.0	36.8
Rajasthan	48.6	47.7	46.1	54.5	53.2	51.7	31.9	23.0	30.5	56.3	53.6	54.3	62.1	59.0	61.5	40.7	29.2	35.7
Uttar Pradesh/ Uttaranchal	44.2	40.2	33.7	46.7	43.5	36.8	36.5	27.4	24.1	54.3	45.1	42.9	57.4	48.6	45.3	44.8	32.2	36.6
Bihar/ Jharkhand	36.0	29.9	21.5	36.3	32.4	21.8	34.0	23.6	20.1	44.2	33.1	23.8	44.7	35.6	25.5	42.4	27.2	14.8
Assam	53.9	44.5	29.7	55.4	48.7	28.3	46.3	42.0	36.5	66.1	48.9	38.2	67.7	54.3	40.6	58.0	45.8	28.8
West Bengal	61.3	52.1	48.8	64.7	54.7	49.1	53.8	46.3	47.1	63.8	57.1	52.6	68.3	61.3	54.6	56.5	49.6	48.3
Odisha	24.3	31.3	29.5	25.2	32.5	31.8	19.9	26.9	22.3	27.5	57.5	26.5	27.6	38.3	27.5	26.9	32.2	24.3
Madhya Pradesh/ Chhattisgarh	38.7	33.4	28.5	42.7	39.3	29.7	28.3	22.4	23.4	46.6	39.2	39.1	50.3	44.6	43.4	37.7	29.4	21.4
Gujarat	29.1	31.2	23.0	32.0	35.5	27.9	24.8	18.3	16.6	40.0	42.0	40.5	44.7	47.4	47.5	32.5	23.3	29.2
Maharashtra	16.2	12.4	13.8	19.2	11.7	11.1	13.0	13.6	16.6	21.1	45.1	22.3	24.9	18.7	21.5	16.6	16.7	23.1
Andhra Pradesh	43.5	33.4	34.3	47.0	35.1	35.0	35.0	27.1	32.3	51.6	41.8	48.5	55.0	44.3	51.0	41.9	32.0	41.3
Karnataka	34.0	28.8	28.6	35.8	29.7	32.6	30.9	25.1	22.4	42.7	37.7	39.0	45.3	39.7	46.2	38.3	27.9	26.4
Goa	20.3	12.0	9.2	23.7	11.4	9.0	17.5	13.4	9.2	34.3	21.1	15.8	43.4	21.8	17.9	27.0	19.6	15.5
Kerala	32.0	39.5	32.4	33.8	42.4	35.0	27.2	27.3	25.4	38.2	47.9	37.1	41.0	51.1	38.9	31.0	34.6	34.2
Tamil Nadu	25.8	21.9	22.1	25.2	22.5	22.3	26.4	20.9	22.0	28.3	26.4	30.0	28.6	27.7	29.3	28.0	23.4	29.1
North East	54.1	52.7	57.2	55.7	53.3	60.7	49.2	50.3	47.2	63.6	57.5	62.2	65.8	58.1	67.0	57.6	55.3	48.6
INDIA TOTAL	37.3	34.6	29.8	41.1	37.2	32.1	29.2	28.3	24.9	43.7	40.5	38.2	47.9	43.8	41.6	35.1	32.5	30.8
**Women**																		
INDIA TOTAL	1.4	2.2	2.7	1.7	1.6	3.4	0.9	0.9	0.3	2.4	3.7	5.0	2.8	2.8	6.2	1.4	1.4	0.8

* *Note: The 2004 estimates are age-specific prevalence estimates from SRSBL. SRSBL provided appropriate age specific patterns that are close to 2011 census population which is used as the standard population for age standardized rates using SFMS and GATS.*

Web Table 2c: The 1998 and 2010 age standardized smoking prevalence for ages 60-69 years and 70+ years, by state and location of residence.

States/ Regions	60-69 years						70+ years					
	Total		Rural		Urban		Total		Rural		Urban	
	1998	2010	1998	2010	1998	2010	1998	2010	1998	2010	1998	2010
**Men**												
Himachal Pradesh	60.5	41.6	62.4	47.4	39.3	8.6	54.7	47.2	56.5	49.4	30.3	0.0
Punjab	10.3	21.6	10.6	25.6	9.6	13.6	8.6	12.2	9.3	16.5	6.6	7.1
Chandigarh	18.6	29.4	24.2	66.7	18.1	26.9	14.6	28.4	26.6	100	13.6	3.9
Haryana	62.6	52.2	72.7	60.6	34.3	46.0	54.8	56.1	64.5	66.8	27.7	16.9
Delhi	17.5	57.8	35.2	35.4	16.7	57.4	11.8	0.0	32.1	0.0	10.9	0.0
Rajasthan	55.0	30.2	59.3	35.8	40.9	18.8	44.6	30.2	50.2	33.3	30.0	42.4
Uttar Pradesh/ Uttaranchal	50.5	45.7	52.9	49.7	40.6	30.3	40.6	32.2	43.1	30.0	31.3	45.1
Bihar/ Jharkhand	42.1	24.4	42.4	25.5	40.6	17.5	33.3	24.8	33.9	24.8	29.2	25.6
Assam	62.9	33.8	65.9	34.9	46.5	35.7	55.3	33.3	58.0	36.4	41.5	0.0
West Bengal	53.2	42.8	59.5	38.7	41.9	48.9	33.4	28.0	39.7	35.3	24.8	16.9
Odisha	23.0	14.7	23.1	14.4	22.1	21.3	17.8	15.6	18.6	20.4	13.1	0.0
Madhya Pradesh/ Chhattisgarh	42.9	37.0	45.3	34.7	34.4	49.4	31.2	31.9	35.4	36.3	19.3	7.4
Gujarat	40.5	35.4	44.2	46.3	32.5	22.5	33.1	47.1	37.6	58.4	23.8	30.1
Maharashtra	19.0	20.2	23.2	24.5	12.7	15.3	13.3	16.9	17.0	21.1	8.4	7.9
Andhra Pradesh	44.8	51.8	48.2	57.7	32.2	36.0	34.3	39.8	37.5	40.7	22.5	37.4
Karnataka	36.0	26.0	38.5	31.1	31.6	18.4	27.0	45.6	30.1	56.6	22.0	16.5
Goa	34.5	10.9	41.5	14.7	27.9	5.0	26.5	3.6	29.8	5.3	23.2	0.0
Kerala	29.5	31.2	31.2	37.8	24.8	12.2	18.4	29.3	19.0	31.2	16.4	22.6
Tamil Nadu	19.9	24.5	20.9	30.5	18.4	19.0	11.4	29.2	12.1	41.6	10.5	16.2
North East	60.2	56.8	62.3	61.3	53.1	41.1	52.7	55.0	54.6	60.9	46.7	17.7
INDIA TOTAL	39.1	33.4	43.0	36.8	28.7	24.8	30.0	30.0	34.1	33.8	19.9	18.7
**Women**												
INDIA TOTAL	3.0	8.7	3.5	10.8	1.8	1.1	2.8	10.6	3.3	12.8	1.6	1.5

Web Table 3: The 1998 and 2010 age standardized rates and age standardized rates ratios of smoking among men aged 15-69 years, by education level and type of product.

Smoke Type	Illiterate or no formal education		Less than 10th grade		10th grade and above	
	**1998/2010**	**ASRR**	**1998/2010**	**ASRR**	**1998/2010**	**ASRR**
**Any Smoking**						
15-29 years	13.9/22.1	1.6 (1.1-2.0)	9.4/14.5	1.5 (1.4-1.7)	4.9/7.5	1.5 (1.3-1.7)
30-44years	46.5/39.9	0.9 (0.8-0.9)	36.0/32.2	0.9 (0.8-1.0)	25.5/20.1	0.8 (0.7-0.9)
45-59years	51.4/47.9	0.9 (0.9-1.0)	41.7/41.2	1.0 (0.9-1.1)	30.1/23.1	0.8 (0.7-0.9)
60-69years	45.1/40.9	0.9 (0.8-1.0)	34.6/32.1	0.9 (0.8-1.1)	22.9/20.2	0.9 (0.6-1.1)
15-69years	33.9/35.4	1.0 (1.0-1.1)	25.1/26.1	1.0 (1.0-1.1)	17.8/15.2	0.9 (0.8-0.9)
70+years	35.2/35.5	1.0 (0.9-1.2)	24.2/28.9	1.2 (1.0-1.4)	15.5/11.2	0.7 (0.3-1.0)
**Cigarettes**						
15-29 years	1.6/10.8	6.8 (2.9-9.8)	2.0/8.1	4.1 (3.6-4.5)	2.0/6.2	3.1 (2.6-3.4)
30-44years	4.3/10.7	2.5 (2.1-2.8)	7.8/14.1	1.8 (1.6-2.0)	12.5/14	1.1 (1.0-1.2)
45-59years	3.7/11.8	3.2 (2.4-3.8)	8.2/15.0	1.8 (1.6-2.0)	15.3/14.7	1.0 (0.8-1.1)
60-69years	2.2/9.3	4.2 (3.0-5.1)	5.3/9.3	1.8 (1.2-2.2)	9.2/11.8	1.3 (0.9-1.6)
15-69years	2.9/10.4	3.6 (3.0-4.0)	5.1/11.2	2.2 (2.1-2.3)	8.5/10.6	1.3 (1.2-1.3)
70+years	1.6/6.8	4.4 (2.7-5.6)	3.2/8.4	2.7 (1.6-3.5)	5.9/3.8	0.6 (0.1-1.0)
**Bidis**						
15-29 years	10.9/14	1.3 (0.9-1.6)	6.6/8.1	1.2 (1.0-1.5)	2.4/1.9	0.8 (0.5-1.0)
30-44years	37.2/33.1	0.9 (0.8-1.0)	25.4/22.7	0.9 (0.8-1.0)	11.0/7.8	0.7 (0.6-0.8)
45-59years	40.2/38.7	1.0 (0.9-1.1)	29.4/30.2	1.0 (0.9-1.1)	12.2/11.1	0.9 (0.7-1.1)
60-69years	32.9/33.4	1.0 (0.8-1.2)	24.4/25.8	1.1 (0.9-1.2)	10.4/11	1.0 (0.5-1.5)
15-69years	26.6/27.9	1.0 (1.0-1.1)	17.7/18.0	1.0 (1.0-1.1)	7.8/6.1	0.8 (0.7-0.9)
70+years	22.4/26.8	1.2 (1.0-1.4)	15.9/21.1	1.3 (1.0-1.6)	6.9/7.4	1.1(0.2-1.6)
