# Supplementary material for: Trends in bidi and cigarette smoking in India from 1998 to 2015, by age, gender and education
Source: BMJ Glob Health. 2016 Apr 6;1(1):e000005. doi: 10.1136/bmjgh-2015-000005 (PMC5321300; doi:10.1136/bmjgh-2015-000005)
Supplement: Supplementary figures [file bmjgh-2015-000005supp_figures.pdf]

Web Figure 1: Age standardized smoking rates among men by age group, product and number of smokers (in millions) in rural India: 1998-2010.

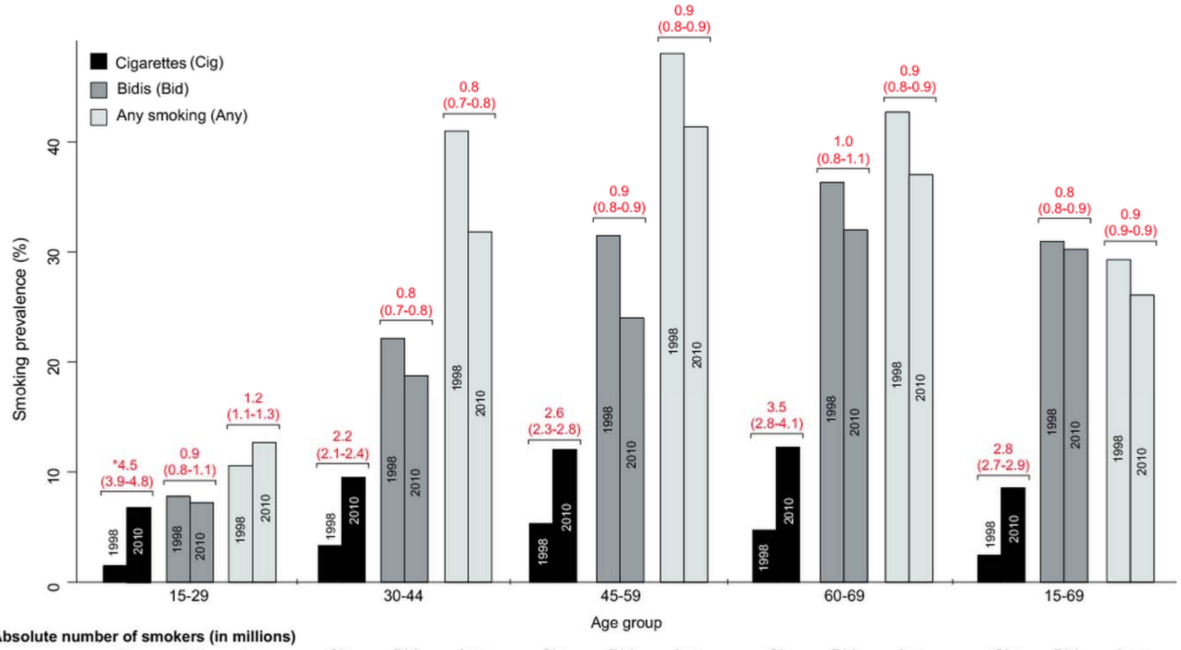

Absolute number of smokers (in millions)

	Cig	Bid	Any	Cig	Bid	Any	Cig	Bid	Any	Cig	Bid	Any	Cig	Bid	Any
**1998**	1.4	7.3	9.7	3.6	20.9	27.3	1.8	14.0	18.5	0.4	5.1	7.0	7.1	46.1	61.0
**2015** Any	11.0	8.5	16.9	14.4	19.9	27.0	9.7	19.0	24.6	2.7	7.1	8.2	37.4	54.5	76.7
Exclusive	7.7	5.8		8.4	13.8		5.9	15.0		1.5	5.4		23.4	40.1	
Both (Cig+Bid)	3.4			4.6			3.2			1.1			12.2		

Note: ASRR=ASR GATS/ ASR SFMS (99% CI)

Web Figure 2: Age standardized smoking rates among men by age group, product and number of smokers (in millions) in urban India: 1998-2010

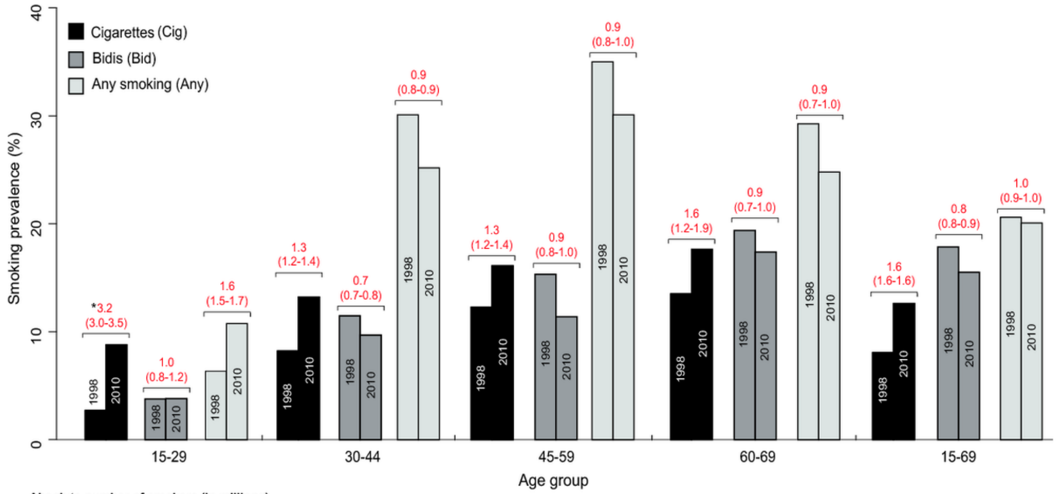

Absolute number of smokers (in millions)

	Cig	Bid	Any	Cig	Bid	Any	Cig	Bid	Any	Cig	Bid	Any	Cig	Bid	Any
**1998**	1.1	1.5	2.8	3.6	4.5	8.6	2.2	3.1	5.7	0.4	0.9	1.5	7.4	10.2	18.7
**2015** Any	7.3	2.4	8.2	8.5	4.7	11.1	6.2	5.3	9.3	1.8	1.8	2.8	23.8	14.1	31.4
Exclusive	4.9	0.8		6.4	2.7		4.3	3.4		1.2	1.0		16.7	8.0	
Both (Cig+Bid)	2.3			1.9			1.6			0.5			6.4		

Note: ASRR=ASR GATS/ ASR SFMS (99% CI)
